# Supplementary material for: Systematic analysis of low-affinity transcription factor binding site clusters in vitro and in vivo establishes their functional relevance
Source: Nat Commun. 2022 Sep 7;13:5273. doi: 10.1038/s41467-022-32971-0 (PMC9452512; doi:10.1038/s41467-022-32971-0)
Supplement: Supplementary file 1 — Supplementary Information [file 41467_2022_32971_MOESM1_ESM.pdf]

Supplementary Information for:
Systematic analysis of low-affinity transcription factor binding site clusters *in vitro* and *in vivo* establishes their functional relevance

Amir Shahein[1], Maria López-Malo[1], Ivan Istomin[1], Evan J. Olson[1], Shiyu Cheng[1], and Sebastian J. Maerkl*[1]

[1]Institute of Bioengineering, School of Engineering, École Polytechnique Fédérale de Lausanne, Lausanne, Switzerland

*Correspondence: sebastian.maerkl@epfl.ch

Supplementary figures and tables

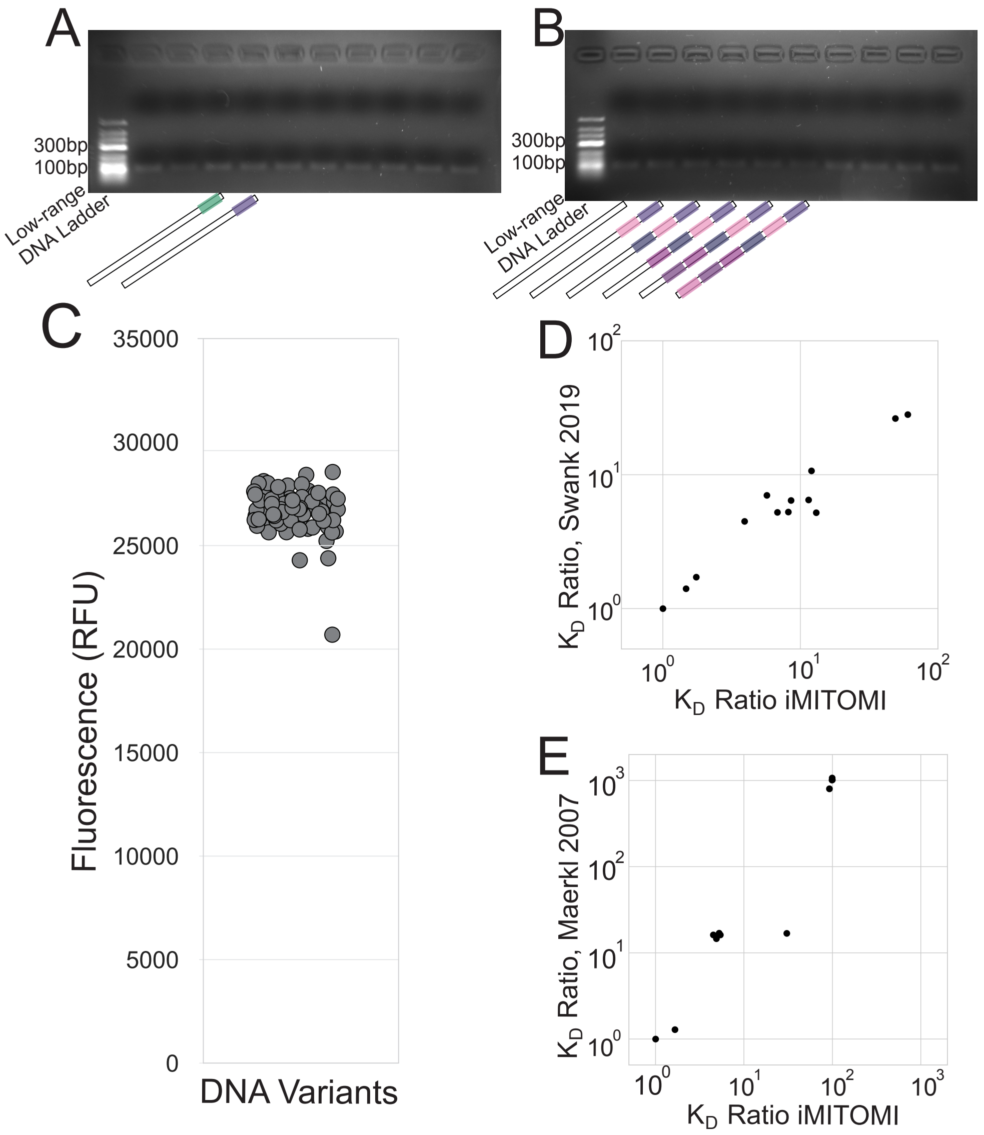

Figure 1: **(A, B)** Agarose gels for iMITOMI 90 bp PCR products corresponding to the multi-weak Zif268 DNA target library characterized in (Figure 2C). A single gel lane was run for each DNA target. **(C)** Fluorescence measurements of concentration-equalized iMITOMI DNA targets in a spotting plate before spotting. **(D, E)** Similar affinities and specificities were obtained when single site DNA targets were characterized on iMITOMI for binding to Zif268, and Pho4, as compared to (1) and (2), respectively. The K_D Ratio represents the K_D divided by the K_D of the strongest binding site analyzed in this study. The observed K_Ds measured for Pho4 appeared to be lower for iMITOMI than the original MITOMI measurement, likely because of the changes in assay geometry used with DNA immobilized on the surface in iMITOMI.

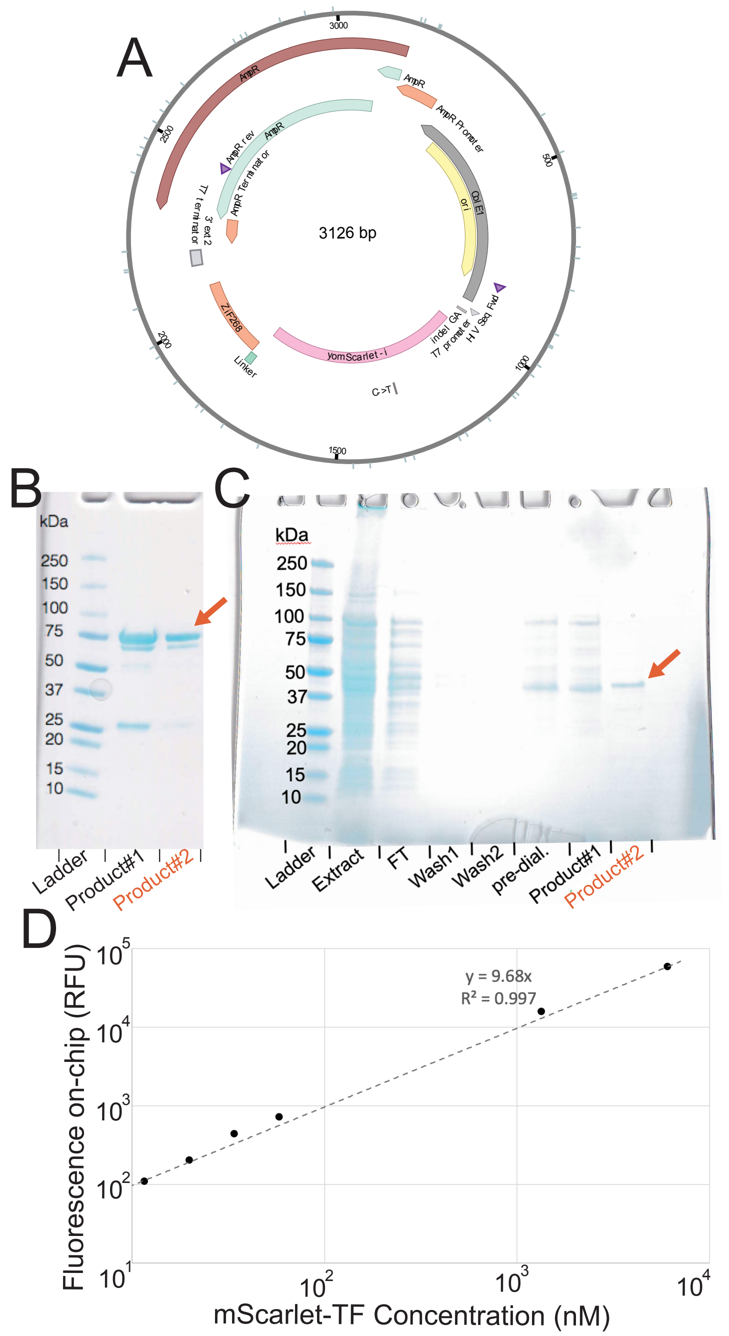

Figure 2: Characterization and calibration for purified proteins. Caption on the next page.

Figure 2: **(A)** Plasmid for 5'-mScarlet-Zif268-6xHis-3' construct under a T7 promoter. A similar construct was used for the Pho4 transcription factor. **(B, C)** Proteins were his-tag purified and ran on a denaturing PAGE gel for Pho4 (**B**) and Zif268 **(C)**. **(B)** Compared to Product #1, Product #2 was additionally buffer exchanged in an Amicon spin-column. **(C)** Product #2 is from an entirely different purification batch. In both cases the higher purity Product #2 was used for all iMITOMI experiments in this study. A gel was run for each purification. **(D)** Calibration curve relating fluorescence measurements on-chip to mScarlet-transcription factor concentration quantitated by a Bradford assay.

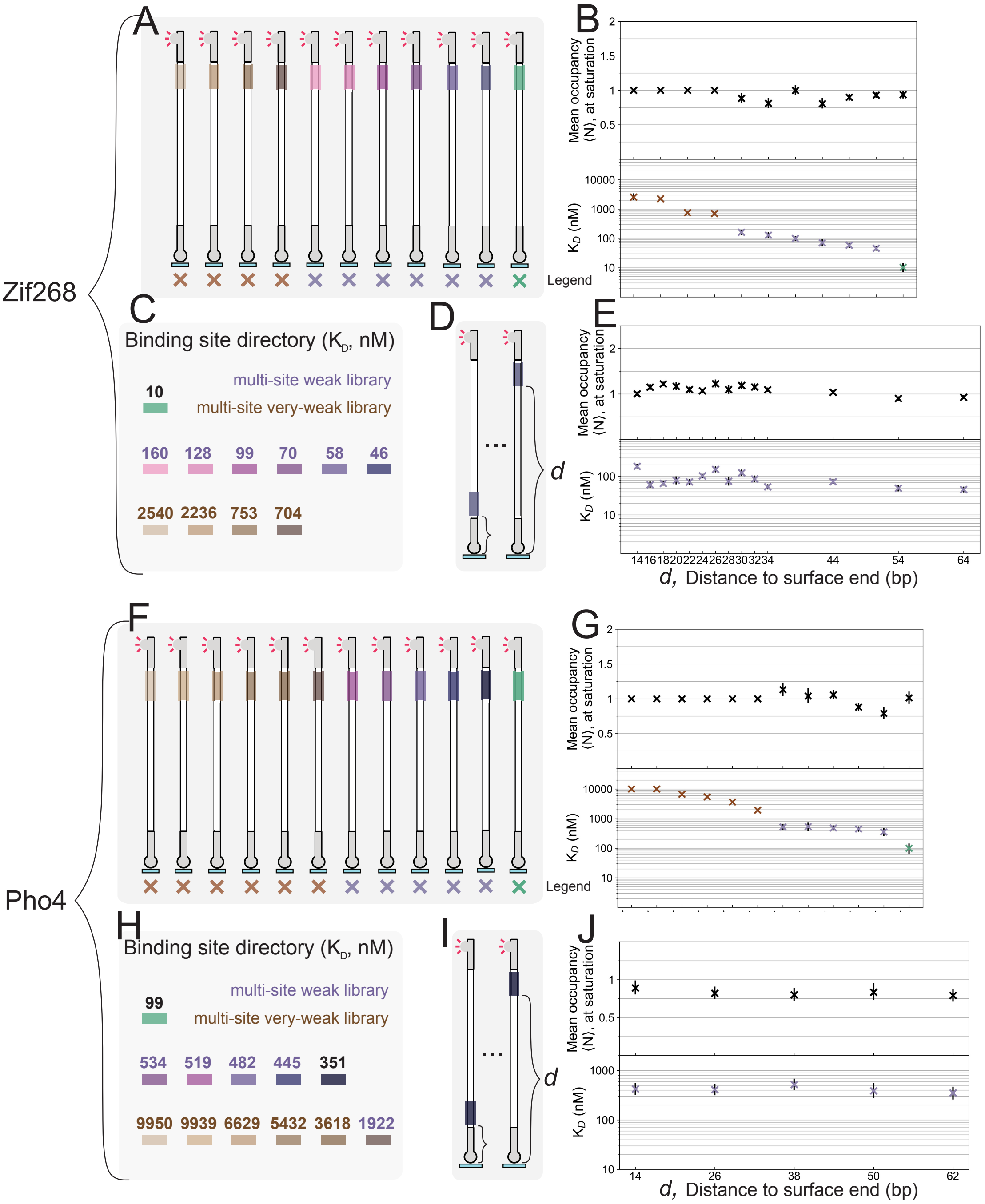

Figure 3: Binding site directories and single site positions scans.

Caption on the next page.

Figure 3: **(A)** Directory: single site DNA targets for binding sites used to build Zif268 clusters throughout the study. **(B)** Mean occupancies at saturation (upper) and K_D values (lower) for targets in **(A)**, in the same order from left to right. For single weak binding sites, the $\langle N \rangle_{\max}$ parameter was fixed. **(C)** Color-coded Zif268 binding site directory with affinity values in nanomolar. **(D)** DNA targets corresponding to the position scan in **(E)**, where a Zif268 weak binding site's distance (d) from the surface coupled end of the DNA was varied across targets in the library. **(E)** Characterization data for the Zif268 single site position scan library. **(F)** Single site DNA targets for binding sites used to build Pho4 clusters throughout the study. **(G)** Mean occupancies at saturation and K_D values for targets in **(F)**. **(H)** Directory: single site DNA targets for binding sites used to build Pho4 clusters throughout the study. **(I)** DNA targets corresponding to a Pho4 binding site position scan. **(J)** Characterization data for the Pho4 position scan library. Similarly to **(E)**, the distance to the chip's surface did not significantly impact binding. K_d and $\langle N \rangle_{\max}$ parameter markers are centered at the mean, while error bars represent 5 to 95% confidence intervals. Samples were analyzed in 18-84 independent chambers, across 2-6 independent experiments.

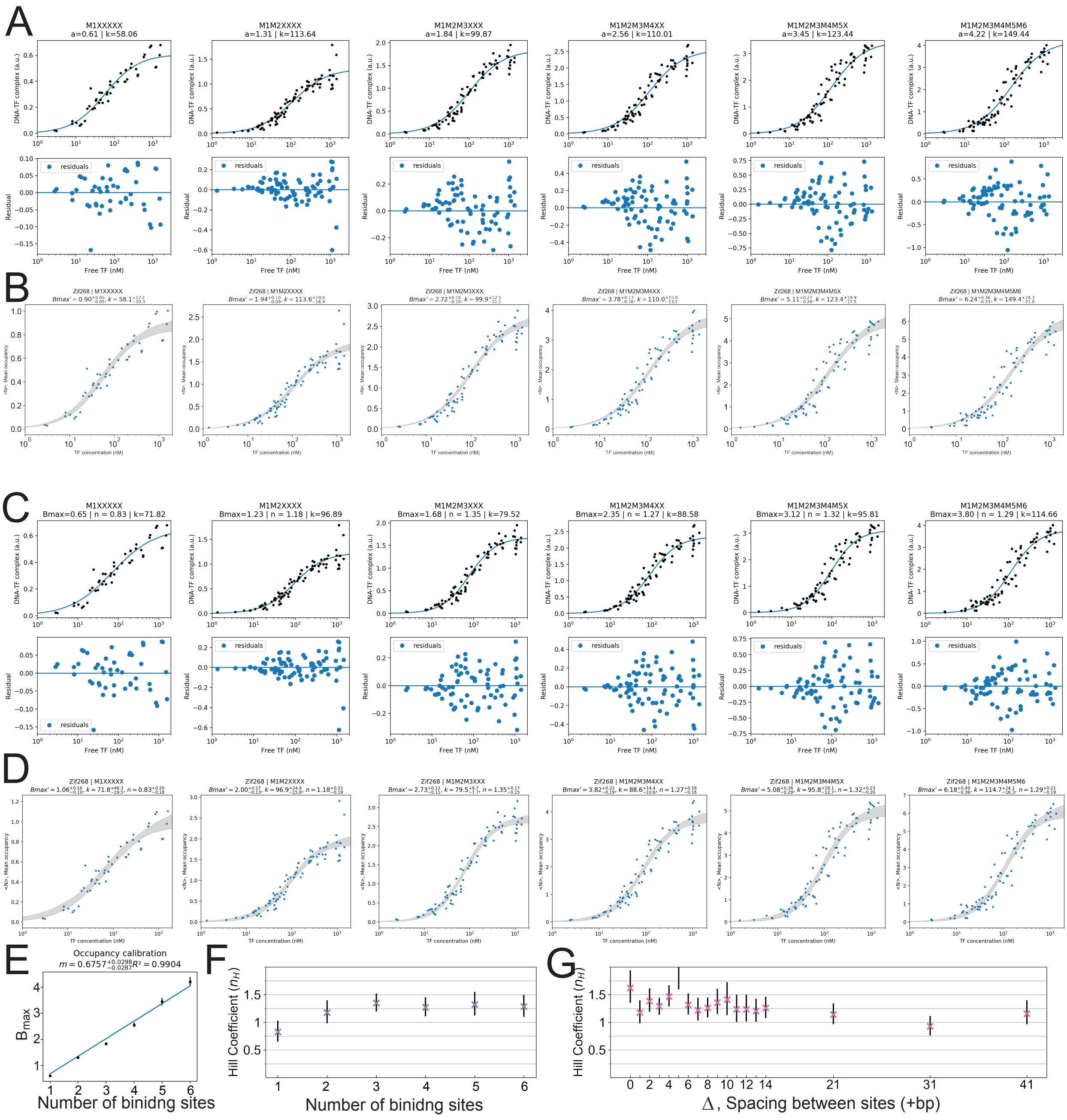

Figure 4: Data and model fits (SBC, Hill) for the Zif268 multi-weak library in Figure 2. Caption on the next page.

Figure 4: **(A)** Data was fit with a 2-parameter saturation binding curve (upper) (Equation 7). Residuals for the fit (lower). **(B)** Parameter space was explored using Markov chain Monte Carlo (MCMC) to obtain a 5 to 95% confidence interval range. Data is normalized by the step increase in B_{max} (Equation 9). **(C, D)** Similar plots to **(A, B)** instead using a Hill model (8). **(E)** Step increase in B_{max} with additional binding sites for the saturation binding model fits in **(A)**. **(F, G)** Hill coefficients (n_H) for the multi-site weak library in **(C, D)** and for the two-site gap-scan library targets (Figure 3) fit with a Hill model. K_d and $\langle N \rangle_{\rm max}$ parameter markers are centered at the mean, while error bars represent 5 to 95% confidence intervals. Samples were analyzed in 18-84 independent chambers, across 2-6 independent experiments.

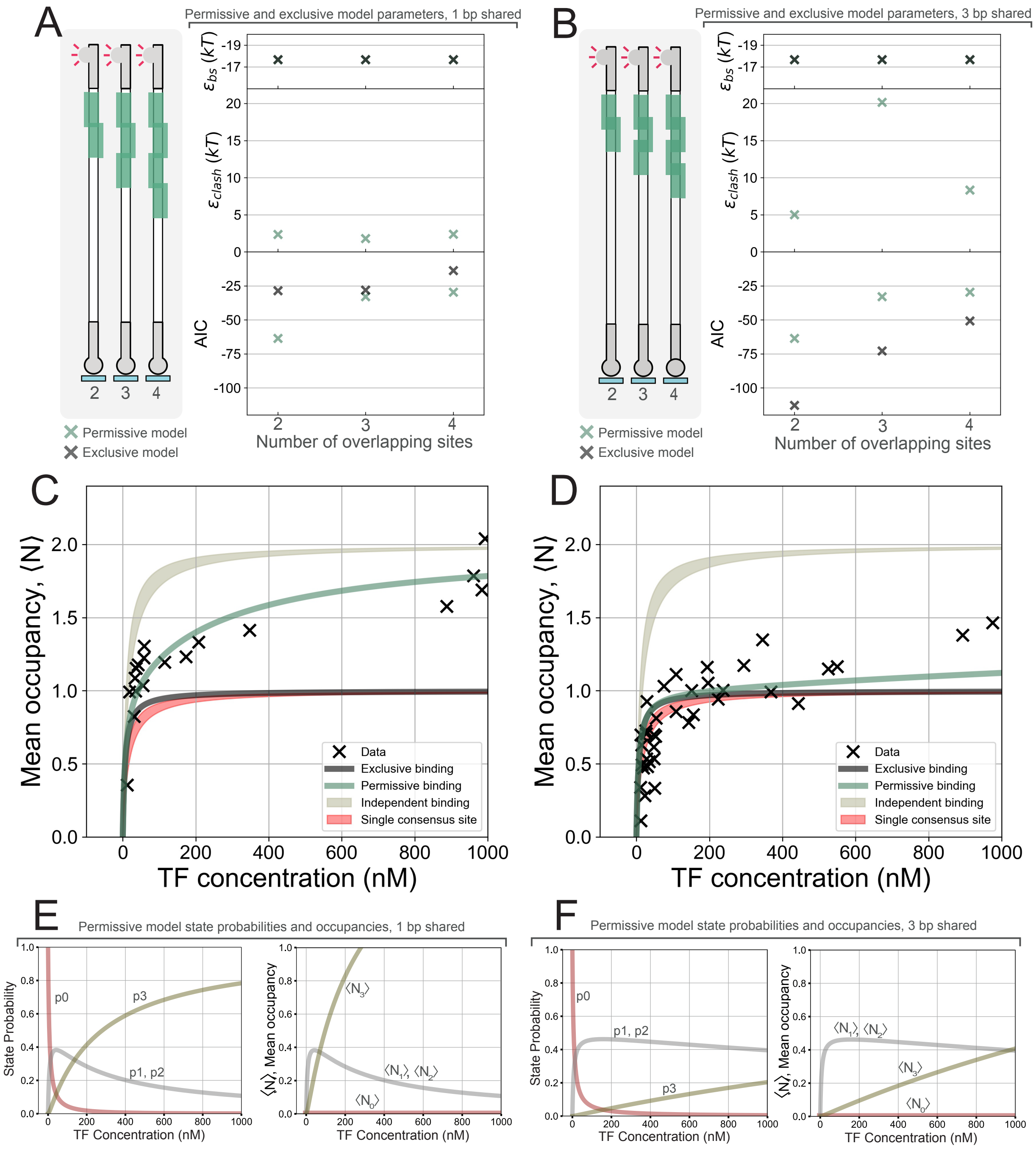

Figure 5: Modeling of binding to high-density clusters.

Caption on the next page.

Figure 5: (**A**) DNA targets for high-density clusters with 1 shared basepair betweeen neighboring consensus binding sites (left). Corresponding parameters and Akaike information criteria (AIC) for exclusive and permissive models of binding (right). Energies are reported in units of kT (product of Boltzmann constant and temperature). (**B**) DNA targets (left) and model parameters (right) for high-density clusters with 3 shared basepairs between neighboring consensus binding sites. **(C)** Characterization data for the DNA target in (**A**) with two consensus binding sites sharing 1 basepair. Exclusive and permissive model predictions in grey and green respectively. 95% confidence intervals for the characterized single consensus binding site (red), and a model of independent binding to two consensus binding sites (beige). (**D**) Corresponding data and models for the DNA target in **(B)** with two consensus binding sites sharing 3 basepairs. (**E, F**) State probabilities and occupanices for the permissive model, for the 2 consensus site DNA targets with 1 shared basepair **(E)** and with 3 shared basepairs **(F)**. K_d and $\langle N \rangle_{\max}$ parameter markers are centered at the mean.

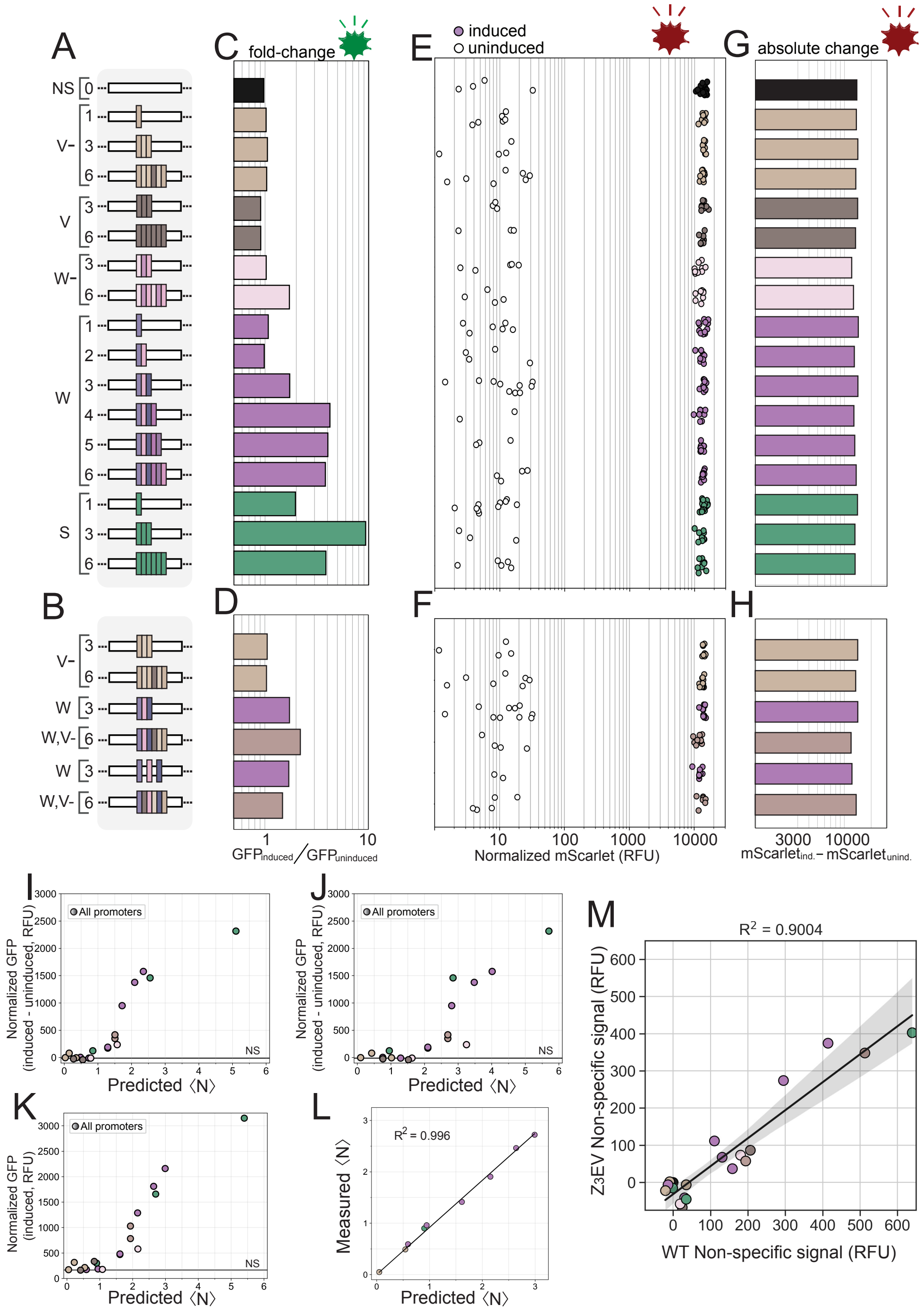

Figure 6: Alternate analyses and control data for the Zif268 *in vivo* results in Figures 5 and 6. Caption on the next page.

Figure 6: **(A, B)** Legends for the characterized minCYC1 promoter libraries. **(C, D)** Alternate data analysis to Figure 5G and 5H, quantifying the Zif268-specific impact on gene expression as GFP mean fold-change between induced and uninduced strains. **(E, F)** Induced and uninduced mScarlet expression levels driven by the control promoter. **(G, H)** Absolute difference between induced and uninduced mScarlet expression levels. **(I, J)** Alternate data analysis to Figure 6, varying the transcription factor concentration from 73 nM (85% consensus saturation) **(I)** to 245 nM (95% consensus saturation) **(J)**. **(K)** Changing the measure of specific gene expression from the difference between mean induced and mean uninduced GFP signal, to mean induced GFP signal. **(L)** Relationship between predicted mean occupancy and measured mean occupancy, for the *in vitro*-characterized DNA targets that were used in the Zif268 *in vivo* study. Predictions were based on an independent statistical thermodynamic binding model similar to Equation 24, but with a variable number of binding sites, parameterized by data collected through characterization of single-site DNA targets, as explained in equation . R^2 of 0.996. **(M)** Relationship between the non-specific signal for the Z_3EV strains containing the Z_3EV transcription factor, and the WT (BY4741) strain containing no transcription factors with a Zif268 DNA binding domain. In each case the non-specific signal was quantified as the absolute mean difference between a given promoter's GFP expression and the GFP expression of the nonspecific (NS) promoter containing no binding sites (similar to Figure 5I and 5J), both in the respective strain's background. R^2 of 0.9, suggesting that the expression in uninduced strains is due to an endogenous transcription factor. Shaded region represents 95% confidence range of the fit.

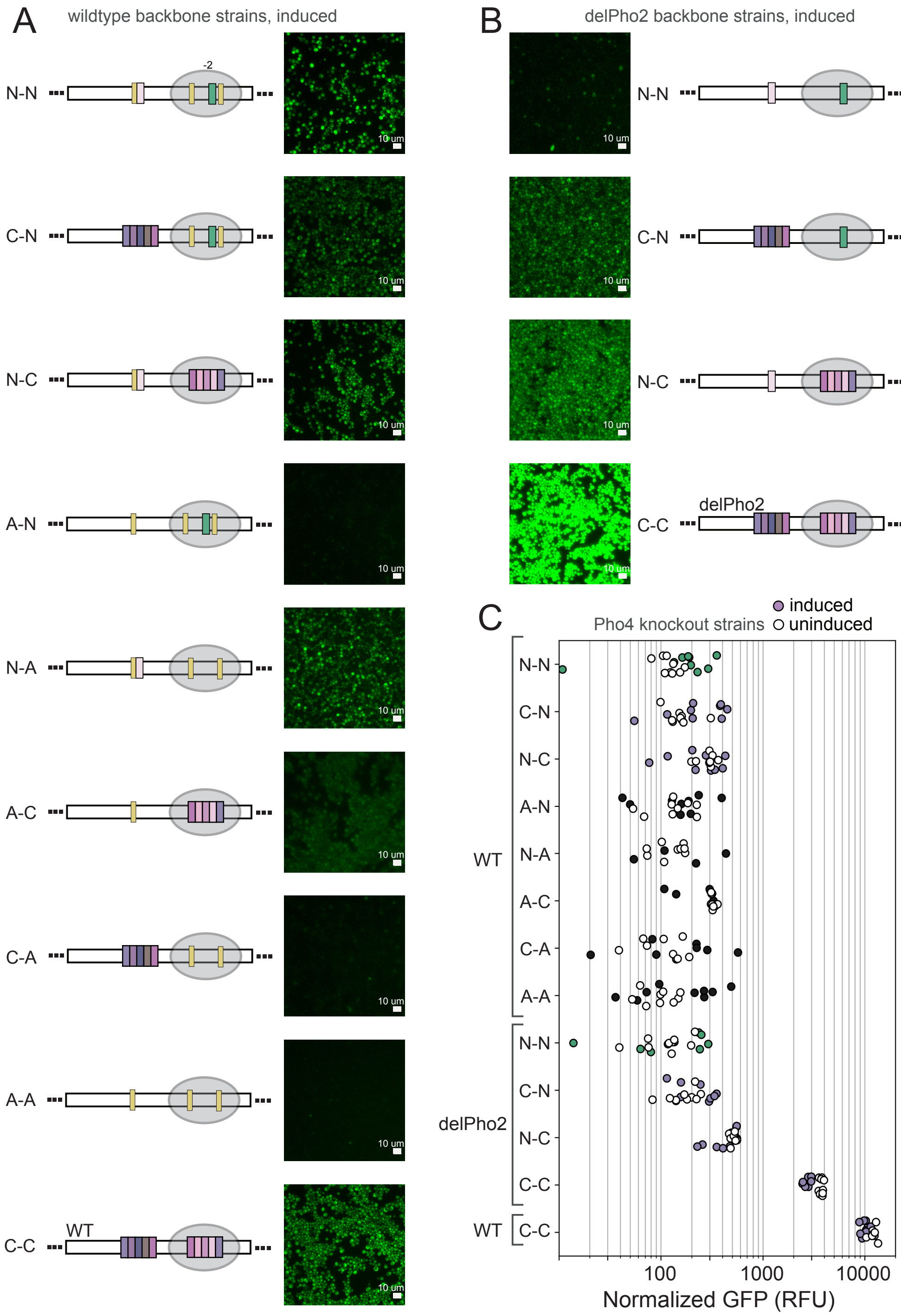

Figure 7: Fluorescence microscope images for the BY4741 strain library, and induced and uninduced platereader measurements for the Pho4 knockout strains.

Caption on the next page.

Figure 7: **(A)** Fluorescence microscope images of the BY4741 strains with wildtype PHO5 promoter backbones induced by phosphate starvation (scalebars represent 10 microns, experiment repeated 2 times with similar results). **(B)** Fluorescence microscope images of the BY4741 strains with delPho2 (all Pho2 sites ablated) PHO5 promoter backbones induced by phosphate starvation. **(C)** Platereader measurements following induction or in the absence of induction, for Pho4 knockout strains, enlarged from Figure 7L. Platereader data was processed similarly to the other platereader measurements in Figure 7.

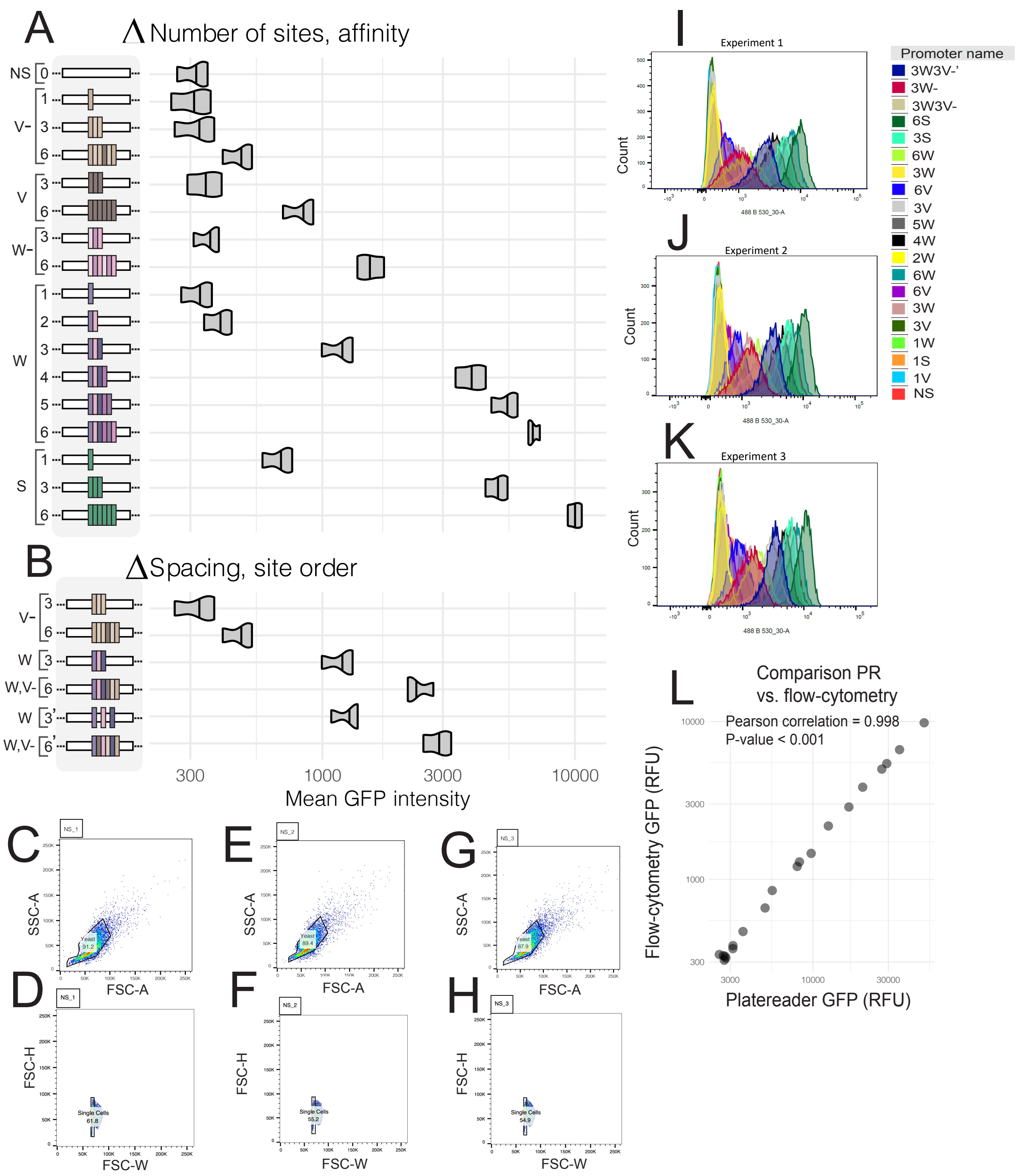

Figure 8: FACS characterization.

Caption on the next page.

Figure 8: Flow-cytometry data, corresponding to the Zif268 *in vivo* study in Figure 5. **(A)** Violin plots describing the distribution of single cell fluorescence intensities, across 3 independent experiments, for a library of strains varying their number of binding sites and affinities. **(B)** Varying promoter spacing and site ordering. **(C-H)** Gating plots for three different sample replicates (non-specific promoter strains). **(I-K)** Fluorescence distributions for the strains tested across three independent experiments. **(L)** Near perfect correlation between platereader (Figure 5) and flow cytometry fluorescence data, r=0.998, P-value < 0.001. The p-value corresponds to a Pearson correlation test (two sided t-test, 18 degrees of freedom, t=34.73).

Reagent	Initial conc.	Volume to add (per 50 μL rxn)
Biotin-tagged primer	100 μM	0.25 μL
Cy5-tagged primer	100 μM	0.25 μL
90bp ssDNA target primer	500 nM	0.25 μL
DreamTaq Green PCR Master Mix	2X	25 μL
Invitrogen UltraPure Distilled Water		24.25 μL

Table 1: iMITOMI targets' PCR recipe

Step	Temperature	Duration
1.	95°C	1 minute
2.	95 °C	30 seconds
3.	50°C	30 seconds
4.	72°C	1 minute
Steps 2 to 4 are cycled a total of 30 times		
5.	72°C	10 minutes
6.	4°C	∞

Table 2: iMITOMI targets' PCR cycling conditions

**Component**	**Final conc.**
**Buffer A**	
NH4Cl	1 M
HEPES	50 mM
MgCl	10 mM
B-ME (fresh)	7 mM
**Buffer B**	
Imidazole	0.5 M
HEPES	50 mM
MgCl	10 mM

KCl	100 mM
B-ME (fresh)	7 mM
**HT buffer**	
HEPES	50 mM
MgCl	10 mM
KCl	100 mM
B-ME (fresh)	7 mM
**HT stock buffer**	
HEPES	50 mM
MgCl	10 mM
KCl	100 mM
Glycerol	60%
B-ME (fresh)	7 mM

Table 3: Purification buffer recipes

2

**Ingredient**	**Amount**
**Nitogen source**	
Ammonium sulfate	5 mg
**Carbon Source**	
Dextrose	20 g
**Amino acids**	
Adenine	130 mg
L-Arginine	50 mg
L-Aspartic Acid	80 mg
L-Histidine HCl	20 mg
L-Isoleucine	50 mg

L-Leucine	100 mg
L-Lysine HCl	50mg
L-Methionine	20 mg
L-Phenylalanine	50 mg
L-Threonine	100 mg
L-Tryptophan	100mg
Uracil	20 mg
L-Tyrosine	50 mg
L-Valine	140 mg
**Vitamins**	
Biotin	2 μg
Calcium Pantothenate	0.4 mg
Folic Acid	2 μg
Inositol	2 mg
Niacin	0.4 mg
P-Aminobenzoic Acid	0.2 mg
Pyridoxine Hydrochloride	0.4 mg
Riboflavin	0.2 mg
Thiamine Hydrochloride	0.4 mg
**Trace elements**	
Boric Acid	0.5 mg
Copper Sulfate	0.04 mg
Potassium Iodide	0.1 mg
Ferric Chloride	0.2 mg
Manganese Sulfate	0.4 mg
Sodium Molybdate	0.2 mg
Zinc Sulfate	0.4 mg
**Salts**	
Magnesium Sulfate	0.5 mg

Calcium Chloride	0.1 mg
Potassium chloride	550 mg
Sodium chloride	100 mg

Table 4: SC PF medium recipe (per Litre)

3

Ingredient	Amount
**Nitogen source**	
Ammonium sulfate	5 mg
**Carbon Source**	
Dextrose	20 g
**Amino acids**	
Adenine	130 mg
L-Arginine	50 mg
L-Aspartic Acid	80 mg
L-Histidine HCl	20 mg
L-Isoleucine	50 mg
L-Leucine	100 mg
L-Lysine HCl	50mg
L-Methionine	20 mg
L-Phenylalanine	50 mg
L-Threonine	100 mg
L-Tryptophan	100mg
Uracil	20 mg
L-Tyrosine	50 mg
L-Valine	140 mg
**Vitamins**	
Biotin	2 μg

Calcium Pantothenate	0.4 mg
Folic Acid	2 μg
Inositol	2 mg
Niacin	0.4 mg
P-Aminobenzoic Acid	0.2 mg
Pyridoxine Hydrochloride	0.4 mg
Riboflavin	0.2 mg
Thiamine Hydrochloride	0.4 mg
**Trace elements**	
Boric Acid	0.5 mg
Copper Sulfate	0.04 mg
Potassium Iodide	0.1 mg
Ferric Chloride	0.2 mg
Manganese Sulfate	0.4 mg
Sodium Molybdate	0.2 mg
Zinc Sulfate	0.4 mg
**Salts**	
Magnesium Sulfate	0.5 mg
Calcium Chloride	0.1 mg
Potassium chloride	550 mg
Sodium chloride	100 mg

Table 5: SC PF medium recipe (per Litre)

4

Supplementary Note 1: Platform development

In our assay, 90 bp-long double stranded DNA (dsDNA) is immobilized on the chip's surface, whereas protein is maintained free in solution and titrated across different regions of the chip (Figure 1D-H, Supplementary Figure 1). This is in contrast to the original MITOMI assay in which transcription factor was immobilized to the surface and 30 bp-long target DNA was added in solution. Inverting the assay chemistry was important to study binding site clusters for several reasons. Using surface-immobilized DNA allows for multiple transcription factor molecules to bind and the resulting increase in fluorescence to be measured, whereas binding of multiple immobilized transcription factors to individual free molecules of DNA would present spatial constraints, and could lead to skewed affinity measurements due to avidity.

The iMITOMI chip contains 768 unit cells, each consisting of a DNA spotting chamber, into which DNA targets are introduced by spotting during fabrication, and a MITOMI detection area, wherein binding occurs and detection is conducted (Figure 1D-F). The spotting chamber and detection area can be separated from each other by pressurizing a neck valve, and a sandwich valve can be pressurized to isolate the individual reaction chambers. A circular MITOMI button valve lies above each detection area, and can be used to mechanically trap molecules bound to the surface in the detection area when this valve is pressurized.

The DNA in each unit cell is individually programmable with a DNA spotter, and fluid flow to each row of unit cells in the chip can be controlled through pressurization of a combination of eight multiplexing valve control lines. We first generate a specific surface chemistry, and immobilize Cy5-tagged dsDNA from each spotting chamber under the button valve in the corresponding detection area before imaging the chip to quantify the surface-bound DNA (Figure 1D). We then flow recombinant mScarlet-tagged transcription factor into the chip (Supplementary Figure 2), using a different concentration in each of the eight pairs of rows. After the DNA and protein are allowed to bind and reach equilibrium, we image the chip to quantify the concentration of free protein at equilibrium in each chamber. Then, by pressurizing the button valve to isolate DNA-bound protein, and after washing away free protein, we image the chip once more to quantify the amount of protein that was in complex with DNA at equilibrium. We can thus directly measure the equilibrium concentrations of free as well as bound protein. We then use these measurements to generate binding curves (Figure 1H) by relating the bound protein signal (normalized by the DNA signal) to

the free protein signal at equilibrium. For each DNA target, a Markov-Chain Monte Carlo sampler is used to fit a 2-parameter saturation binding curve model.

To validate our method, we conducted several experiments to ascertain that the affinities and specificities measured with iMITOMI correspond to previous MITOMI measurements, which were validated against data obtained with other methods generally considered a gold standard (3; 2; 4). Affinity and specificity measurements taken for single binding site targets were found to be consistent with previous results obtained using standard MITOMI across a wide affinity range, both for Zif268 (1; 5) and Pho4 (2) (Supplementary Figure 1D-E). To test whether using surface-bound DNA might influence transcription factor binding, we placed single binding sites for Zif268 and Pho4 in different positions of the DNA target. No difference in either K_D or B_max was observed as the binding site is moved closer towards the chip surface (Supplementary Figure 3), indicating that the distance from the chip surface did not introduce systematic differences in the ability of a transcription factor to bind to the target DNA strand.

Supplementary Note 2: Data analysis and modeling

iMITOMI data analysis and modeling

Image analysis and data processing

Images from a given scan were stitched together using ImageJ and ROIs were processed using GenePix, which facilitated automatic and manual feature alignment, as well as conversion of ROI pixel intensities to mean signal intensities.

Quantification of free and bound protein signals

A normalized bound signal intensity was quantified as:

$$S_{bound} = \frac{TF_{bound} - TF_{background}}{DNA_{immobilized} - DNA_{background}} \tag{1}$$

S_{bound} corresponds to the bound transcription factor at equilibrium (background corrected) normalized by the amount of DNA at equilibrium (background corrected). The values used in this calculation were mean signal intensities from an ROI under the button valve, at the appropriate assay stage discussed in section Methods: iMITOMI experiments.

A signal S_{free} corresponding to free protein at equilibrium was quantified using an ROI next to the button valve as:

$$S_{free} = TF_{free} - TF_{background} \tag{2}$$

This signal was calibrated against the calibration curves from Figure 2 to obtain absolute TF concentrations, [TF].

Saturation binding curve parameter estimation

According to mass action kinetics, at equilibrium a 1:1 transcription factor (TF) to binding site (BS) binding reaction follows the relationship

$$[\text{TF}] + [\text{BS}] \underset{\text{k}_{off}}{\overset{\text{k}_{on}}{\rightleftharpoons}} [\text{TF:BS}]$$

$$k_{on} \cdot [TF][BS] = k_{off} \cdot [\text{TF:BS}]$$

$$K_D = \frac{k_{off}}{k_{on}} = \frac{[TF][BS]}{[\text{TF:BS}]}$$

$$[\text{TF:BS}] = \frac{[TF][BS]}{[K_D]} \tag{3}$$

Furthermore, the proportion of binding sites bound by transcription factor molecules, P_{bound}, can be defined as follows

$$P_{bound} = \frac{[\text{TF:BS}]}{[\text{TF:BS}] + [BS]} \tag{4}$$

Through substituting Equation 3 into Equation 4 and rearranging, it follows that

$$P_{bound} = \frac{[TF]}{K_D + [TF]} \tag{5}$$

This derives a relationship to model the proportion of binding sites bound by transcription factor, based on the concentration of free transcription factor and the binding reaction's dissociation constant (K_D, representing the affinity).

The same functional form as in Equation 5 can be used similarly to describe a binding reaction to DNA targets containing multiple binding sites, if all binding site affinities are assumed equivalent, and binding is assumed to be independent. To see this, it is useful to consider that [BS] was used in

the above analysis instead of [DNA], and the analysis is not sensitive to if some constant number of [BS] are distributed on fewer molecules of DNA. The B_{max} parameter represents the fluorescence intensity at transcription factor concentrations for a given DNA target, and using it to scale P_{bound} allows for a rescaling from proportion of binding sites bound to S_{bound}, that is

$$S_{bound} = P_{bound} \cdot B_{max} \tag{6}$$

Therefore, experimental data characterizing saturation binding to a given DNA target can be fit according to the two parameter (K_D, B_{max}) model in equation 7

$$S_{bound} = \frac{B_{max} \cdot [TF]}{K_D + [TF]} \tag{7}$$

where [TF] is in absolute concentration units (nM) to obtain a K_D estimate in nM. As discussed, B_{max} represents the fluorescence level at saturating transcription factor concentrations.

In order to model cooperative binding, a common phenomenological modification to Equation 7 is the incorporation of the Hill coefficient (n_H), even for 1:1 binding reactions, which produces the Hill model:

$$S_{bound} = \frac{B_{max} \cdot [TF]^{n_H}}{K_D + [TF]^{n_H}} \tag{8}$$

For a given DNA target, data were combined across two to six experimental days and an initial fit to Equation 7 was obtained through non-linear least squares minimization with the Lmfit package in Python (6). To explore the parameter space around this fit initial parameter set and obtain confidence intervals, we used Markov Chain Monte Carlo to conduct sampling of the posterior probability distribution given the experimental data and assuming a uniform prior (Figure 4B and C).

To obtain mean occupancies $\langle N \rangle$, S_{bound} (units RFUs) was normalized (calibrated) according to the B_{max} increase from adding additional binding sites (or equivalently, the RFUs per 1 TF molecule bound to DNA on average), as described in the results section of the main text. This converts equation 7 to the more interpretable form in equation 9

$$\langle N \rangle = \frac{n_{sites} \cdot [TF]}{K_D + [TF]} \tag{9}$$

where the mean occupancy $\langle N \rangle$ represents the average number of transcription factor molecules bound per DNA molecule, and n_{sites} represents the number of defined binding sites on the DNA

target. Upon normalization, B_{max} on the right hand side is converted to n_{sites} since the B_{max} of a given target can be thought of as the B_{max} from each binding site, multiplied by the number of binding sites.

Statistical mechanical modeling

Statistical mechanical models of binding were built starting from the useful framework developed by Philips et. al. and presented in the Physical Biology of the Cell textbook (7). We recommend referring to the textbook for a detailed discussion of the modeling framework. Essentially, microstates of the binding system were grouped into states, where each state represents all of the possible microstates of the system where a particular set of DNA binding sites are bound by transcription factor molecules (e.g. the unbound state represents all microstates of the system where the DNA is not bound by any transcription factor molecule). Here a microstate signifies a particular distinguishable arrangement of indistinguishable transcription factor molecules among positions of a spatially discretized environment (e.g. the unbound state is composed of many microstates, which differ from one another based on which positions in the lattice-modeled solution are occupied). Each state can be attributed a total energy, by accounting for the energies from all particles in one of its microstates. This total energy does not differ between the different microstates of a given state. Furthermore, each state is attributed a multiplicity, by counting the number of different microstates that can give rise to the state.

In order to calculate the probability of any state, first recognize that

$$p_i \propto e^{-E_i/(kT)}$$

$$\beta = \frac{1}{kT}$$

$$p_i \propto e^{-\beta E_i} \tag{10}$$

where p_i is the probability of microstate i, E_i is the total energy from all particles in microstate i, and the constant kT is a product of the Boltzmann constant k and the absolute temperature T and usually written as β. The term $e^{-\beta E_i}$ is the Boltzmann factor. Equation 11 relates the ratio of probabilities for two different microstates to the ratio of their Boltzmann factor's, which depends

on the difference between their total energies. This captures the typical intuition that lower-energy configurations are more favorable.

$$\frac{p_i}{p_j} = e^{\beta(E_j - E_i)} \tag{11}$$

To determine the probability for a given microstate (Equation 12), its Boltzmann factor must be normalized by the sum of the Boltzmann factors from every possible microstate (the partition function, Z, Equation 13), such that the microstate probabilities sum to 1.

$$p_i = \frac{e^{-\beta E_i}}{Z} \tag{12}$$

$$Z = \Sigma_i \; e^{-\beta E_i} \tag{13}$$

In order to calculate the probability of any given state (group of microstates), it follows that the state's multiplicity can be multiplied by the state's Boltzmann factor. This produces what is effectively the sum over Boltzmann factors from all microstates belonging to the state, a term referred to as the state's weighted multiplicity (7), which accordingly when normalized by the partition function produces the state's probability.

For illustration, it is useful to first consider how to count the number of microstates (how to calculate the multiplicity) for the unbound state

$$\frac{\Omega!}{L!(\Omega - L)!} \tag{14}$$

Where Ω represents the number of discrete positions where a transcription factor could be located in solution, and L represents the number of transcription factor molecules. Transcription factor molecules can be placed in solution in $\frac{\Omega!}{(\Omega - L)!}$ different configurations, but they can be rearranged indistinguishably $L!$ ways.

Furthermore, the following related approximation will enable us to simplify our analysis

$$\frac{x!}{y!(x-y)!} \approx \frac{x^y}{y!}, \quad \text{if } x \gg y \tag{15}$$

More generally, state multiplicites evaluate to

$$\frac{\Omega!}{(L-n)! \; (\Omega - (L-n))!} \tag{16}$$

Where n represents the number of TF molecules bound to the DNA molecule

Which according to Approximation 15 simplifies to

$$\frac{\Omega^{L-n}}{(L-n)!} \tag{17}$$

Since we assume that in our binding system $\Omega \gg L$

Table 6 and Equations 18 to 25 demonstrate how to model a simple system of transcription factor molecules binding to DNA having a single binding site, in order to illustrate how the model in Equation 5 that we derived from mass action kinetics can be equivalently derived through statistical mechanics. This connection ultimately allowed us to coherently integrate both mass action kinetic and statistical mechanical modeling in our study.

State	Energy, E_i	Multiplicity	Relative weighted multiplicity
—	$L \cdot \varepsilon_{sol}$	$\frac{\Omega!}{L!(\Omega-L)!} \approx \frac{\Omega^L}{L!}$	1
$\underline{O}$	$\varepsilon_{bs} + (L-1) \cdot \varepsilon_{sol}$	$\frac{\Omega!}{(L-1)!(\Omega-(L-1))!} \approx \frac{\Omega^{L-1}}{(L-1)!}$	$\frac{[TF]}{[TF]_o} \cdot e^{-\beta \Delta \varepsilon_{bs}}$

Table 6: 1:1 binding through the lens of statistical mechanics

The total energy, E_i, of the unbound state (_) is signified by the sum of an ε_{sol} energy term from each of the L particles in solution. For the bound state ($\underline{O}$), one particle less will be at an energy of ε_{sol}, and instead will have the energy ε_{bs}, representing its specific interaction to a single binding site on DNA. Multiplicities are weighted according to their Boltzmann factors as follows, for the unbound state the weighted multiplicity is

$$\frac{\Omega^L}{(L)!} \cdot e^{-\beta L \varepsilon_{sol}} \tag{18}$$

And for the bound state, the weighted multiplicity is

$$\frac{\Omega^{L-1}}{(L-1)!} \cdot e^{-\beta((L-1)\varepsilon_{sol}+\varepsilon_{bs})} \tag{19}$$

Furthermore, following the convention in (7), we normalize the weighted multiplicities and partition function by the weighted multiplicity of the unbound state, to represent these terms as relative weighted multiplicities (Table 6, Equation 20). For the bound state the relative weighted multiplicity simplifies to

$$\frac{\frac{\Omega^{L-1}}{(L-1)!} \cdot e^{-\beta((L-1)\varepsilon_{sol}+\varepsilon_{bs})}}{\frac{\Omega^{L}}{(L)!} \cdot e^{-\beta L \varepsilon_{sol}}}$$

$$= \frac{L}{\Omega} \cdot e^{-\beta(\varepsilon_{bs}-\varepsilon_{sol})} \tag{20}$$

Furthermore, as illustrated in (7),

$$[TF] = \frac{L}{\Omega \cdot V_{box}}$$

$$[TF]_o = \frac{1}{V_{box}}$$

$$\frac{[TF]}{[TF]_o} = \frac{L}{\Omega} \tag{21}$$

where [TF] is the concentration of transcription factor, and the constant $[\mathrm{TF}]_o$ is a reference concentration that corresponds to if each position in solution was occupied by a transcription factor molecule (7). Furthermore, V_{box} is the volume of one discrete lattice position in solution. This allows the bound state's relative multiplicity to be expressed as

$$\frac{[TF]}{[TF]_o} \cdot e^{-\beta \Delta\varepsilon_{bs}}, \quad \text{where } \Delta\varepsilon_{bs} = \varepsilon_{bs} - \varepsilon_{sol} \tag{22}$$

Then, the probability of any state can be determined by normalizing by the partition function (expressed relative to the unbound state) Z, which is the sum of all relative weighted multiplicities

$$Z = 1 + \frac{[TF]}{[TF]_o} \cdot e^{-\beta \Delta\varepsilon_{bs}} \tag{23}$$

Therefore, the probability of the bound state is

$$p_{bound} = \frac{\frac{[TF]}{[TF]_o} \cdot e^{-\beta \Delta\varepsilon_{bs}}}{1 + \frac{[TF]}{[TF]_o} \cdot e^{-\beta \Delta\varepsilon_{bs}}} \tag{24}$$

We used the single-site binding model in Equation 24, which is equivalent to the expression for mean occupancy for single-site targets, to obtain energy parameters for particular binding sites, by fitting the corresponding single-site DNA targets' binding data (normalized by the increase in

B_{max} with additional sites) to the model (using non-linear least-squares minimization, followed by MCMC, as before). These binding site energies were used to parameterize higher-order statistical mechanical models of binding to DNA targets containing multiple binding sites (e.g. for modeling exclusive and permissive binding to high-density clusters).

Equation 24 is the statistical mechanical equivalent to Equation 5, considering that

$$K_D = [TF]_o \cdot e^{\beta \Delta \varepsilon_{bs}} \tag{25}$$

As discussed, this connection permitted us to move between statistical mechanical and mass action modeling frameworks in our study.

Exclusive binding model to describe binding to high-density clusters

In exclusive binding models, transcription factors were not permitted to be bound simultaneously to overlapping binding sites (sites sharing common basepairs), as illustrated in the main manuscript Figure 4D and Table 7. For example, for a cluster with two binding sites, only three states (unbound, left site bound, or right site bound) were considered in the exclusive model. The states, energies, and multiplicites for an exclusive model of binding to a DNA target containing two binding sites are shown in Table 7.

State	Energy, E_i	Multiplicity	Relative weighted multiplicity
——	$L \cdot \varepsilon_{sol}$	$\frac{\Omega!}{L!(\Omega-L)!} \approx \frac{\Omega^L}{L!}$	1
O—	$\varepsilon_{bs1} + (L-1) \cdot \varepsilon_{sol}$	$\frac{\Omega!}{(L-1)!(\Omega-(L-1))!} \approx \frac{\Omega^{L-1}}{(L-1)!}$	$\frac{[TF]}{[TF]_o} \cdot e^{-\beta \Delta \varepsilon_{bs1}}$
—O	$\varepsilon_{bs2} + (L-1) \cdot \varepsilon_{sol}$	$\frac{\Omega!}{(L-1)!(\Omega-(L-1))!} \approx \frac{\Omega^{L-1}}{(L-1)!}$	$\frac{[TF]}{[TF]_o} \cdot e^{-\beta \Delta \varepsilon_{bs2}}$

Table 7: Exclusive binding model for a high-density cluster with two overlapping sites

In order to calculate the mean occupancy of transcription factors on DNA, we can calculate a probability-weighted average of the number of transcription factors bound in each state. Therefore, for the example of exclusive binding to two overlapping sites (states presented in Table 7)

$$\langle N \rangle = 0 \times \frac{1}{Z} + 1 \times \frac{\frac{[TF]}{[TF]_o} \cdot e^{-\beta \Delta \varepsilon_{bs1}}}{Z} + 1 \times \frac{\frac{[TF]}{[TF]_o} \cdot e^{-\beta \Delta \varepsilon_{bs2}}}{Z}, \quad \text{where} \tag{26}$$

$$Z = 1 + \frac{[TF]}{[TF]_o} \cdot e^{-\beta \Delta \varepsilon_{bs1}} + \frac{[TF]}{[TF]_o} \cdot e^{-\beta \Delta \varepsilon_{bs2}} \tag{27}$$

Permissive binding model to describe binding to high-density clusters

In permissive binding models, transcription factors were permitted to be bound simultaneously to overlapping binding sites, as illustrated in the main manuscript Figure 4D and Table 8. For example, for a cluster with two overlapping binding sites, four states were permitted (unbound, left site bound, right site bound, or both sites bound).

State	Energy, E_i	Multiplicity	Relative weighted multiplicity
__	$L \cdot \varepsilon_{sol}$	$\frac{\Omega!}{L!(\Omega-L)!} \approx \frac{\Omega^L}{L!}$	1
O_	$\varepsilon_{bs1} + (L-1) \cdot \varepsilon_{sol}$	$\frac{\Omega!}{(L-1)!(\Omega-(L-1))!} \approx \frac{\Omega^{L-1}}{(L-1)!}$	$\frac{[TF]}{[TF]_o} \cdot e^{-\beta \Delta \varepsilon_{bs1}}$
_O	$\varepsilon_{bs2} + (L-1) \cdot \varepsilon_{sol}$	$\frac{\Omega!}{(L-1)!(\Omega-(L-1))!} \approx \frac{\Omega^{L-1}}{(L-1)!}$	$\frac{[TF]}{[TF]_o} \cdot e^{-\beta \Delta \varepsilon_{bs2}}$
OO	$\varepsilon_{bs1} + \varepsilon_{bs2} + \varepsilon_{clash} + (L-2) \cdot \varepsilon_{sol}$	$\frac{\Omega!}{(L-2)!(\Omega-(L-2))!} \approx \frac{\Omega^{L-2}}{(L-2)!}$	$(\frac{[TF]}{[TF]_o})^2 \cdot e^{-\beta(\Delta \varepsilon_{bs1} + \Delta \varepsilon_{bs2} + \varepsilon_{clash})}$

Table 8: Permissive binding model for a high-density cluster with two overlapping sites

For binding to larger clusters where two or more transcription factors can be bound at once (i.e. the state with both sites bound in the permissive model in Table 8), this must be taken into account in these states' multiplicities. For illustration, consider the multiplicity of a state with two transcription factors bound at once. According to Equation 16 and Equation 17.

$$\frac{\Omega!}{(L-2)!\ (\Omega-(L-2))!} = \frac{\Omega^{L-2}}{(L-2)!} \tag{28}$$

Which when normalized by the multiplicity of the unbound state results in the relative multiplicity

$$\frac{\frac{\Omega^{L-2}}{(L-2)!}}{\frac{\Omega^L}{L!}} = \frac{L(L-1)}{\Omega^2} \tag{29}$$

The term on the RHS of Equation 29 can be re-written as

$$\frac{\frac{L!}{(L-2)!}}{\Omega^2} \tag{30}$$

Which according to Approximation 15

$$= \frac{L^2}{\Omega^2} \quad if \;\; L \gg 2 > 0 \tag{31}$$

More generally, a state with n transcription factors bound at once will have the relative weighted multiplicity

$$\left(\frac{L}{\Omega}\right)^n, \quad \text{if } L \gg n > 0$$

$$= \left(\frac{[TF]}{[TF]_o}\right)^n \tag{32}$$

Permissive model states possess an additional energy parameter ε_{clash} (Table8, bottom) for each set of shared basepairs (at the junction of two overlapping sites) where two transcription factors are bound simultaneously. For instance, for a cluster with three binding sites where the first site overlaps the second, and the second overlaps the third, the state with three transcription factors bound simultaneously would possess $2 \cdot \varepsilon_{clash}$in its energy term. ε_{clash} can be considered as composed of both an energy associated with steric interference between transcription factor molecules, and some loss of binding to the shared basepairs, lumped into a single parameter.

Model selection

To compare models of binding to high-density clusters, the Akaike Information Criterion (AIC) and Bayesian Information Criterion (BIC) were computed using the Lmfit package (6). The AIC and BIC can be computed as:

$$AIC = N \cdot ln(\frac{\chi^2}{N}) + 2N_{vars} \tag{33}$$

$$BIC = N \cdot ln(\frac{\chi^2}{N}) + ln(N) \cdot N_{vars} \tag{34}$$

$$\tag{35}$$

Where χ^2 is the chi-square, N is the number of data points, and N_{vars} is the number of floating parameters. For clusters where 1 basepair was shared between neighboring binding sites, the lower

AIC and BIC suggest that the permissive model better represents binding than the exclusive model (Figure 5). On the other hand, as the number of shared basepairs increased to 33% of the motif (3bp), binding was better modeled as exclusive rather than permissive, as reflected by the lower AIC and BIC for the exclusive model.

In vivo data analysis and modeling

Modeling independent binding to clusters using parameters derived from single site DNA targets

Our results identified that binding sites with a positive gap distance in lower-density clusters exhibit largely independent binding. Therefore to model binding to these clusters, accounting for the difference in affinities between different sites, we used an independent binding model parameterized by energies derived from characterization of the individual binding sites on single site DNA targets. We used a statistical mechanical model that accounts for the different states of the system, although a similar result could be obtained by summing the occupancy contribution from each individual binding site in isolation.

To predict the mean occupancy of Zif268 clusters *in vivo*, we simulated a concentration range from 85% to 95% saturation of the consensus binding site (73 nM to 245 nM), confirming our results under a wide range of concentrations expected for the Z_3EV transcription factor. To check the accuracy of our model and premise of binding independence, for clusters that we characterized fully *in vitro* we compared our model predictions to measured occupancies (Figure 6M), which showed near perfect agreement and an R^2 value of 0.996.

To quantify induced and uninduced levels of gene expression from platereader timeseries measurements, the signal in the appropriate fluorescence or OD600 channel from blank wells containing media alone was subtracted. Then the fluorescence timeseries measurements were normalized by their corresponding OD600 values. To represent induced and uninduced levels for each replicate, timeseries measurements were averaged across a time window before the strains reached stationary phase, and all data was plotted in strip plots using Seaborn and Matplotlib in Python. Target transcription factor-specific and non-specific signals were then quantified as explained in the results section.

References

[1] Zoe Swank, Nadanai Laohakunakorn, and Sebastian J Maerkl. Cell-free gene-regulatory network engineering with synthetic transcription factors. *Proceedings of the National Academy of Sciences*, 116(13):5892–5901, 2019.

[2] Sebastian J Maerkl and Stephen R Quake. A systems approach to measuring the binding energy landscapes of transcription factors. *Science*, 315(5809):233–237, January 2007.

[3] Chaitanya Rastogi, H Tomas Rube, Judith F Kribelbauer, Justin Crocker, Ryan E Loker, Gabriella D Martini, Oleg Laptenko, William A Freed-Pastor, Carol Prives, David L Stern, et al. Accurate and sensitive quantification of protein-DNA binding affinity. *Proceedings of the National Academy of Sciences*, 115(16):E3692–E3701, 2018.

[4] Marcel Geertz, David Shore, and Sebastian J Maerkl. Massively parallel measurements of molecular interaction kinetics on a microfluidic platform. *Proceedings of the National Academy of Sciences*, 109(41):16540–16545, October 2012.

[5] Matthew C Blackburn, Ekaterina Petrova, Bruno E Correia, and Sebastian J Maerkl. Integrating gene synthesis and microfluidic protein analysis for rapid protein engineering. *Nucleic Acids Research*, 44(7):e68–e68, 2015.

[6] M Newville, T Stensitzki, DB Allen, and A Ingargiola. Lmfit: Non-linear least-square minimization and curve-fitting for python, doi: 10.5281/zenodo. 11813, 2014.

[7] Rob Phillips, Jane Kondev, Julie Theriot, Hernan G Garcia, and Nigel Orme. *Physical biology of the cell.* Garland Science, 2012.
