# Supplementary material for: Systematic analysis of low-affinity transcription factor binding site clusters in vitro and in vivo establishes their functional relevance
Source: Nat Commun. 2022 Sep 7;13:5273. doi: 10.1038/s41467-022-32971-0 (PMC9452512; doi:10.1038/s41467-022-32971-0)
Supplement: Supplementary file 2 — Reporting Summary [file 41467_2022_32971_MOESM2_ESM.pdf]

nature portfolio

Corresponding author(s): Professor Sebastian Maerkl

Last updated by author(s): Feb 14, 2022

Reporting Summary

Nature Portfolio wishes to improve the reproducibility of the work that we publish. This form provides structure for consistency and transparency in reporting. For further information on Nature Portfolio policies, see our Editorial Policies and the Editorial Policy Checklist.

Statistics

For all statistical analyses, confirm that the following items are present in the figure legend, table legend, main text, or Methods section.

n/a	Confirmed	
☐	☒	The exact sample size (*n*) for each experimental group/condition, given as a discrete number and unit of measurement
☐	☒	A statement on whether measurements were taken from distinct samples or whether the same sample was measured repeatedly
☐	☒	The statistical test(s) used AND whether they are one- or two-sided<br>*Only common tests should be described solely by name; describe more complex techniques in the Methods section.*
☒	☐	A description of all covariates tested
☐	☒	A description of any assumptions or corrections, such as tests of normality and adjustment for multiple comparisons
☐	☒	A full description of the statistical parameters including central tendency (e.g. means) or other basic estimates (e.g. regression coefficient) AND variation (e.g. standard deviation) or associated estimates of uncertainty (e.g. confidence intervals)
☒	☐	For null hypothesis testing, the test statistic (e.g. *F*, *t*, *r*) with confidence intervals, effect sizes, degrees of freedom and *P* value noted<br>*Give P values as exact values whenever suitable.*
☐	☒	For Bayesian analysis, information on the choice of priors and Markov chain Monte Carlo settings
☒	☐	For hierarchical and complex designs, identification of the appropriate level for tests and full reporting of outcomes
☐	☒	Estimates of effect sizes (e.g. Cohen's *d*, Pearson's *r*), indicating how they were calculated

Our web collection on statistics for biologists contains articles on many of the points above.

Software and code

Policy information about availability of computer code

Data collection	NIS Elements (Nikon) was used for imaging with a Nikon Ti Eclipse fluorescent microscope.
Data analysis	Genepix Pro 7 was used to aid with image processing. ImageJ 1.51 was used to aid with processing microscope images. Custom code was used for data processing, analysis, and visualization. All custom code has been made available on GitHub (https://github.com/eukaryoting/systematic_analysis_of_low-affinity_clusters), as referenced in the data availability statement in the manuscript. It is presented in Jupyter Notebooks for simple use by other individuals.

For manuscripts utilizing custom algorithms or software that are central to the research but not yet described in published literature, software must be made available to editors and reviewers. We strongly encourage code deposition in a community repository (e.g. GitHub). See the Nature Portfolio guidelines for submitting code & software for further information.

Data

Policy information about availability of data

All manuscripts must include a data availability statement. This statement should provide the following information, where applicable:

- Accession codes, unique identifiers, or web links for publicly available datasets
- A description of any restrictions on data availability
- For clinical datasets or third party data, please ensure that the statement adheres to our policy

Source data are provided with this paper. All data is available in the following GitHub repository: https://github.com/eukaryoting/systematic_analysis_of_low-affinity_clusters, in the source_data folder.

Field-specific reporting

Please select the one below that is the best fit for your research. If you are not sure, read the appropriate sections before making your selection.

- [x] Life sciences
- [ ] Behavioural & social sciences
- [ ] Ecological, evolutionary & environmental sciences

For a reference copy of the document with all sections, see nature.com/documents/nr-reporting-summary-flat.pdf

Life sciences study design

All studies must disclose on these points even when the disclosure is negative.

Sample size	Sample sizes were not pre-determined based on statistical methods. However, methods used were generally high-throughput, resulting in significant amounts of replicates. Furthermore, Bayesian statistics relate sample sizes to a measure of certainty about relationships/findings.
Data exclusions	In Genepix Pro, assay chambers that were not suitable for analysis (noise/high background signal blocking the region-of-interest, or absence of pulled down DNA) were excluded from analysis.
Replication	All experiments were replicated across multiple experimental days. Initial findings were subject to follow-up experiments to challenge or refine understanding, as presented in the manuscript. Details on how many replicates and independent experiments were performed has been written into the Figure captions.
Randomization	Samples were not randomized since this was not deemed important for any particular experiment. Samples were distributed across chips for analysis through a cycling spotting pattern. We conducted data analysis of how experimental parameters (e.g. chip row, plate position, ROI signals) might relate to one another (e.g. corner plots, data exploration/plotting), and found no unexpected relationships/artifacts arising due to the experimental setup.
Blinding	Blinding was not relevant since in the experimental process, samples were processed in batch and generally in an automated way. For example, robotic spotting introduces samples automatically to the chip, which are then processed all together by flowing in each reagent to all chambers at once. During imaging the microscope automatically scans the entire chip. Expression normalization accounts for any possible differences in the plate before sample spotting (DNA samples are also equalized in fluorescence, Figure S1C). Furthermore, during image processing experimenters do not know which chamber corresponds to which sample. The yeast strains were numerous and labeled according to an identifier rather than a descriptive code (and then only re-connected to promoter description during python analysis, using a lookup table).

Reporting for specific materials, systems and methods

We require information from authors about some types of materials, experimental systems and methods used in many studies. Here, indicate whether each material, system or method listed is relevant to your study. If you are not sure if a list item applies to your research, read the appropriate section before selecting a response.

Materials & experimental systems

n/a	Involved in the study	
x		Antibodies
	x	Eukaryotic cell lines
x		Palaeontology and archaeology
x		Animals and other organisms
x		Human research participants
x		Clinical data
x		Dual use research of concern

Methods

n/a	Involved in the study	
x		ChIP-seq
	x	Flow cytometry
x		MRI-based neuroimaging

Eukaryotic cell lines

Policy information about cell lines

Cell line source(s)	BY4741, McIsaac et. al. 2012 (see manuscript).
Authentication	All final cell lines were sequence verified.
Mycoplasma contamination	Cell lines were not tested for mycoplasma contamination.
Commonly misidentified lines (See ICLAC register)	*Name any commonly misidentified cell lines used in the study and provide a rationale for their use.*

Flow Cytometry

Plots

Confirm that:

- [x] The axis labels state the marker and fluorochrome used (e.g. CD4-FITC).
- [x] The axis scales are clearly visible. Include numbers along axes only for bottom left plot of group (a 'group' is an analysis of identical markers).
- [x] All plots are contour plots with outliers or pseudocolor plots.
- [x] A numerical value for number of cells or percentage (with statistics) is provided.

Methodology

Sample preparation	CYC1 minimal promoter strains were grown overnight in SC medium lacking uracil at 30 ºC shaking at 250 rpm. Cultures were diluted at O.D. of 0.175 in fresh SC medium lacking uracil. Cells were grown until log phase, OD ~ 0.8 and then were diluted and induced to starting OD of 0.1 – 0.2 in SC medium lacking uracil with beta-estradiol (Sigma-Aldrich) at 200 nM. Cells were induced for 12 h. Cells were diluted to OD ~ 0.5 in phosphate buffered saline (PBS) to assay fluorescent expression by flow cytometry.
Instrument	BD LSRFortessa
Software	FACSDiva was used to collect flow cytometry data, FlowJo was used to analyze flow cytometry data.
Cell population abundance	N/A
Gating strategy	For all data we acquired 10,000 events. Events were gated by forward (FSC-A) and side scatter (SSC-A) to select the population of interest. Then doublets were excluded gating by FSC-W (Pulse Width (W)) and FSC-H (Pulse Height (H)). Gating plots are provided as Supplementary pdfs. The median of the fluorescent distribution were calculated. Positive and negative GFP and mScarlet controls were used to ensure that the instrument maintained proper calibration.

- [x] Tick this box to confirm that a figure exemplifying the gating strategy is provided in the Supplementary Information.
